# The Role of Radiomics and Artificial Intelligence Applied to Staging PSMA PET in Assessing Prostate Cancer Aggressiveness

**DOI:** 10.3390/jcm14103318

**Published:** 2025-05-09

**Authors:** Luca Urso, Ilham Badrane, Luigi Manco, Angelo Castello, Federica Lancia, Jeanlou Collavino, Alessandro Crestani, Massimo Castellani, Corrado Cittanti, Mirco Bartolomei, Gianluca Giannarini

**Affiliations:** 1 Department of Translational Medicine, University of Ferrara, Via Aldo Moro 8, 44124 Ferrara, Italy; rsulcu@unife.it (L.U.); ilham.badrane@unife.it (I.B.); ctc@unife.it (C.C.); 2 Nuclear Medicine Unit, Onco-Hematology Department, University Hospital of Ferrara, 44124 Ferrara, Italy; m.bartolomei@ospfe.it; 3 Medical Physics Unit, University Hospital of Ferrara, 44124 Ferrara, Italy; luigi.manco@ausl.fe.it; 4 Nuclear Medicine Unit, Fondazione IRCCS Ca’ Granda Ospedale Maggiore Policlinico, 20122 Milan, Italy; massimo.castellani@policlinico.mi.it; 5 Oncology Unit, University Hospital of Ferrara, 44124 Ferrara, Italy; lncfrc@unife.it; 6 Urology Unit, Santa Maria della Misericordia University Hospital, 33100 Udine, Italy; jeanloucollavino@gmail.com (J.C.); alessandro.crest@gmail.com (A.C.); g.giannarini@gmail.com (G.G.); 7 Department of Medicine, University of Udine, 33100 Udine, Italy

**Keywords:** radiomic, radiomic features, artificial intelligence, PET, PSMA, prostate cancer (PCa), staging, biochemical recurrence, prognosis

## Abstract

**Background**: PSMA PET is essential tool in the management of prostate cancer (PCa) patients in various clinical settings of disease. The tremendous growth of the implementation of radiomics and artificial intelligence (AI) in medical imaging techniques has led to an increasing interest in their application in prostate-specific membrane antigen (PSMA) PET. The aim of this article is to systemically review the current literature that explores radiomics and AI analyses of staging PSMA PET towards its potential application in clinical practice. **Methods**: A systematic research of the literature on three international databases (PubMed, Scopus, and Web of Science) identified a total of 166 studies. An initial screening excluded 68 duplicates and 72 articles relevant to other topics. Finally, 21 studies met the inclusion criteria. **Conclusions**: The literature suggests that radiomic analysis could improve the characterization of tumor aggressiveness, the prediction of extra-capsular extension, and seminal vesicles involvement. Moreover, AI models could contribute to predicting BCR after radical treatment. Limitations regarding heterogeneous objectives of investigation, and methodological standardization of radiomics analysis still represent the main obstacle to overcome in order to see these technology break through into daily clinical practice.

## 1. Introduction

Prostate cancer (PCa) ranks as the second most common malignancy and the fifth leading cause of cancer-related mortality among men worldwide [1].

Over the past few decades, the incidence of PCa has consistently increased, primarily due to the widespread use of serum prostate specific antigen (PSA) testing. However, this form of opportunistic screening has led to over-diagnosis of a large number of clinically indolent tumors [2]. To prevent overtreatment of such cases, therapeutic management often involves watchful waiting or active surveillance. Conversely, radical treatment must be offered to patients with non-metastatic clinically relevant PCa. Two main radical treatments are available, which are prostatectomy (preferably robotic) and external beam radiation therapy (RT). In selected cases, RT may be followed by the maintenance of androgen blockade through androgen deprivation therapy (ADT), while surgery may be followed by adjuvant RT [3,4].

Within five years of surgery, 30–50% of patients experience biochemical recurrence (BCR) of PCa [5]. The most widely accepted definition of biochemical recurrence post-surgery is a PSA level of 0.2 ng/mL or higher, confirmed by at least two consecutive rising measurements. For radiotherapy-treated patients, BCR is defined by the “Phoenix” criteria, which identify any PSA value of ≥2 ng/mL above the nadir achieved [6,7]. In case of BCR, systemic treatments or local treatments (i.e., metastases-directed therapy) can be performed [8].

Choosing an individualized therapy tailored to each patient is essential, as it significantly influences the disease’s progression. Employing a multimodal and multidisciplinary diagnostic approach is critical for identifying the most effective and personalized treatment. Such an approach combines the assessment of the mutational profile (genomics), phenotype (proteomics), and imaging modalities including the new and promising radiomics approach.

Radiomics consists of the high-throughput extraction—either automated or semi-automated—of numerous quantitative features from digital medical images, based on the idea that medical images harbor additional information beyond what is perceptible to the human eye. Furthermore, these images capture underlying pathophysiological processes, reflecting genomic and proteomic patterns through macroscopic, image-based features [9].

In nuclear medicine, radiomic analysis has gained particular prominence in the context of positron emission tomography (PET), with several radiotracers, including radiolabeled prostate-specific membrane antigen (PSMA) PET, used in the assessment of PCa patients [10].

Radiomics, through the support of Artificial Intelligence (AI) tools, has the potential to provide extensive in vivo non-invasive insights into the biological characteristics of tumors. This enables the assessment of intra-patient tumor heterogeneity, eliminating the need for repeated biopsies over time and overcoming the inevitable limitation of sampling error associated with biopsies. Such an approach offers a comprehensive, holistic view of the patient’s lesions, thereby supporting a more personalized and appropriate therapeutic decision-making [11].

The aim of this article is to offer a systematic review of the literature about the potential role of radiomic analysis and AI performed on staging PSMA-ligands PET to guide the management of PCa patients.

## 2. Materials and Methods

This systematic review was conducted in compliance with a standardized protocol [12] and according to the “Preferred Reporting Items for a Systematic Review and Meta-Analysis of Diagnostic Test Accuracy Studies” (PRISMA-DTA statement) [13]. The complete PRISMA checklist is in the Supplementary Material (Table S1).

A clear review question was formulated, which included (1) the index test (e.g., radiomic analysis and artificial intelligence applied to staging PET/CT or PET/MRI with PSMA-ligands radiopharmaceuticals), (2) the patient cohort and target disease (e.g., characterization of disease aggressiveness in newly diagnosed PCa patients), and (3) the outcome measures (characterization of the primary tumor, prediction of local extension, prediction of BCR).

Systematic research of the published literature was conducted up to the 3rd of January 2025 by two independent reviewers (L.U. and G.G.). Three international databases were screened (PubMed, Scopus, and Web of Science) using the following keywords: “prostate cancer AND (PSMA OR prostate specific membrane antigen OR prostate-specific membrane antigen) AND (PET or Positron Emission Tomography) AND radiomic* AND (baseline OR diagnosis OR staging)”. Only articles in the English language performing a radiomic analysis of staging PSMA-ligands PET (prior to any radical or systemic treatment) in biopsy-proven newly diagnosed PCa patients were considered. Exclusion criteria were as follows: (a) articles in languages other than English; (b) preclinical articles; (c) articles of a type other than original research articles (i.e., reviews, commentaries, editorials, conference proceedings); original research articles performed in patients with suspicious but not biopsy-proven PCa; original articles performed in patients who had already received any kind of treatment for the PCa; original articles investigating PCa without PSMA expression at PET imaging. Any discrepancy was resolved by consensus. After excluding duplicates, a first screening of title, abstract, and keywords was performed by two independent reviewers (L.U. and I.B.). The full text of articles who passed this first screening were retrieved and further evaluated by the same two reviewers to confirm their eligibility. Two reviewers (L.U. and I.B.) carefully checked the reference list of selected papers to detect any other potentially eligible studies. A meta-analysis could not be performed due to the remarked heterogeneity of the studies identified in the literature.

The following data were collected for each study included in this systematic review: (a) first author; (b) year of publication; (c) study design—retrospective vs. prospective; (d) number of patients included; (e) type of PSMA-ligands radiotracer used; (f) type of imaging modality used—PET/CT vs. PET/MRI; (g) main aim(s); (h) main result(s).

The risk of bias in the included studies was assessed using the Critical Appraisal Skills Programme (CASP) checklist dedicated to diagnostic studies, (https://casp-uk.net/aboutus, accessed on 15 January 2025). The critical appraisal was performed by two independent reviewers (L.U. and I.B.), resolving discrepancies by discussion and consensus. Moreover, two reviewers (L.M. and L.U.) independently performed the radiomics quality score (RQS) according to the model proposed by Lambin et al. [14] to assess the quality of the radiomic analysis in the selected papers. Again, any discrepancies were solved by discussion and consensus.

## 3. Results

**Figure 1:**
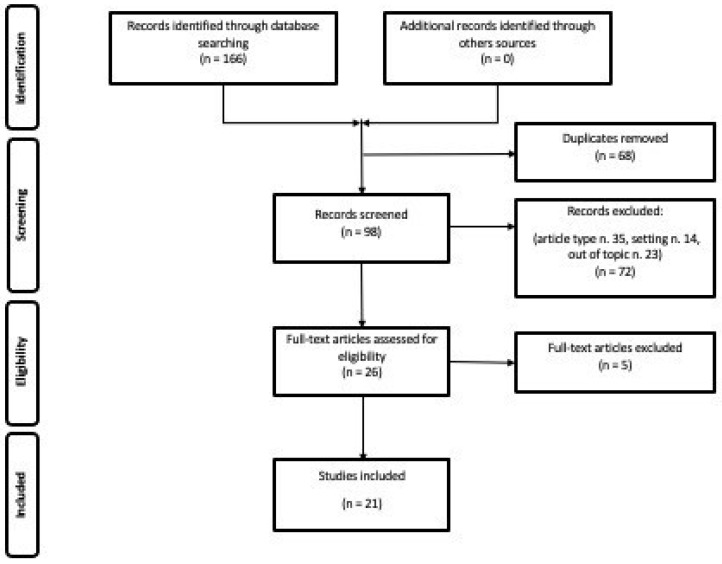
PRISMA flowchart representing the research strategy used to select the articles to include in this systematic review.

Overall, 21 original research articles met the inclusion criteria and were included in this systematic review. Figure 1 depicts the Preferred Reporting Items for Systematic Reviews and Meta-analysis (PRISMA) research strategy.

The articles included were all published after 2019 (Table 1). Six (28.6%) studies had a prospective design, whereas most were retrospective (n = 15; 71.4%). A total of 2497 patients were analyzed across the studies selected. Three different PSMA-ligands were used in the studies retrieved: [^68^Ga]Ga-PSMA-11 in 12 papers (57.1%), [^18^F]F-PSMA-1007 in 7 papers (33.3%), and [^18^F]F-DCFPyL in 2 (9.5%). Moreover, in one study, [^18^F]F-choline was used as radiotracer along with [^68^Ga]Ga-PSMA-11, although it was not considered for radiomic analysis [15]. Radiomic analysis was performed on different imaging modalities. In particular, eight papers analyzed only PET imaging, seven focused on PET/CT, and six on both PET and magnetic resonance imaging (MRI), executed in a hybrid scanner or in two separate scanners.

**Figure 2:**
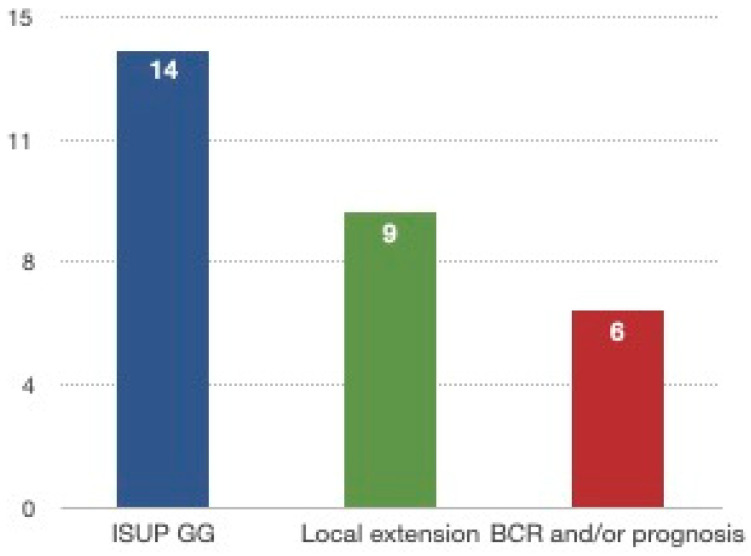
The distribution of the studies according to their objective of investigation.

Overall, the articles were organized in three groups according to their objective of investigation. The first group included articles aiming to predict the International Society of Urological Pathology (ISUP) grade group of newly diagnosed PCa and included 14 studies and 1304 patients. Nine studies aimed to predict adverse pathologic features of the disease in 978 patients. Finally, prediction of BCR and prognosis was the aim of six studies that enrolled a total of 770 patients. Of note, eight studies [15,16,17,18,19,20,21] had multiple aims and were classified in two groups each. The distribution of the studies and the cumulative number of patients enrolled stratified by macro-area is represented in Figure 2.

### 3.1. Bias Analysis

**Figure 3:**
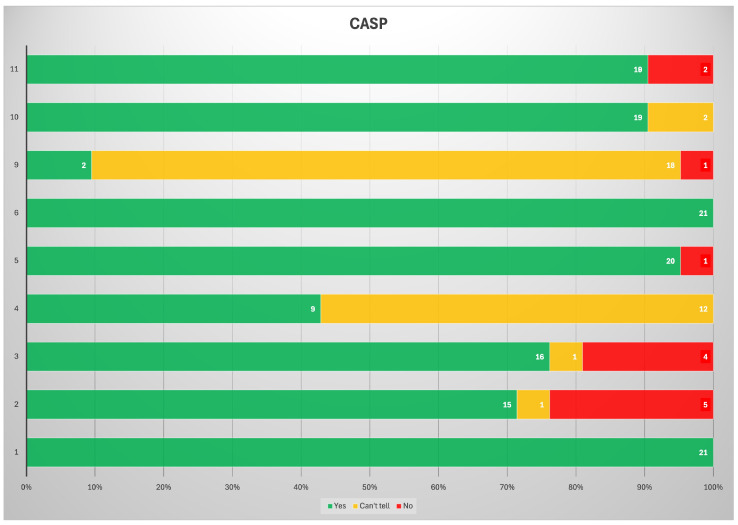
Results of the CASP analysis of the studies included.

According to the CASP analysis, the risk of bias was low (Figure 3). Two main biases were identified in the studies. First, for most of the studies (57.1%), the influence of the results of the reference standard on those of the study could not be ruled out. This was due to either retrospective study design or absent warranty of experimenters blinded to the standard of reference. Second, most of the results published still cannot be translated to other patient populations. This is still one of the main limits of radiomic analysis and artificial intelligence applied to medical imaging. Most of the studies are retrospective (71.4%) and involve a single center (90.5%). Therefore, they lack external validation and harmonization of radiomics features. Nevertheless, each of the remaining seven items totalized a score of more than 70%. Therefore, the methodological quality of the studies considered is globally high.

### 3.2. RQS

**Figure 4:**
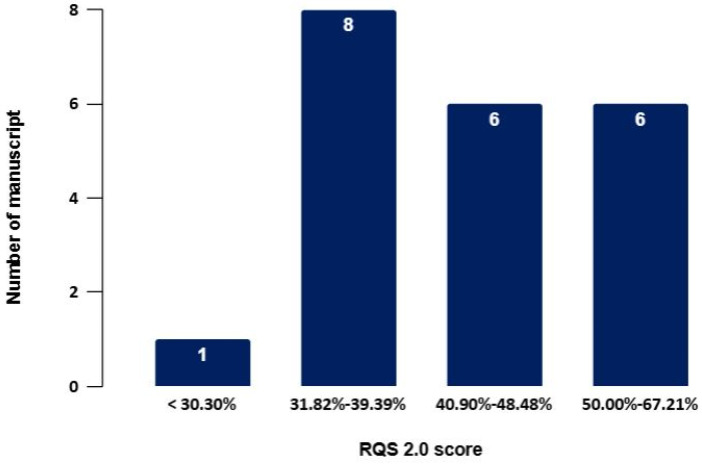
Histograms of average RQS scores according to the blinded analysis of two authors.

The RQS values for the included studies ranged from 20 (30.30%) to 41 (67.21%) (Table 2 and Table 3). As illustrated in Figure 4, 28% of the analyzed studies obtained a score above 40%. Studies with scores exceeding 40% but remaining below 50% often exhibited methodological limitations that constrained their overall quality assessment [15,16,21,22,23], indicating a radiomics methodology that partially satisfies the criteria established by Lambin et al. [14]. Six out of twenty-one papers achieved an RQS score exceeding 50%. Specifically, five of these studies were assessed using the conventional radiomics approach [24,25,26,27,28], while only one study utilized the deep learning radiomics methodology [29].

### 3.3. Characterization of the Primary Tumor Prediction of Adverse Pathologic Features of the Primary Tumor

In the first work published with this aim, Zamboglou et al. [16] identified a radiomic feature extracted from [^68^Ga]Ga-PSMA-11 PET (gray level size zone matrix—GLSZM) able to predict both the primary tumor aggressiveness (ISUP grade group ≥ 3 vs. <3) and the lymph node involvement (pN0 vs. pN1) with optimal areas under the curve (AUC = 0.91 and 0.85, respectively). The prediction of the ISUP grade group by radiomic features was successfully achieved also by Feliciani et al. [22] using radiomics extracted from both apparent diffusion coefficient (ADC) sequence in MRI and [^68^Ga]Ga-PSMA-11 PET. Similarly, Solari et al. [30] investigated the same aim in 101 patients staged with [^68^Ga]Ga-PSMA-11 PET/MRI. The authors performed a radiomic analysis on both sets of images (MRI and PET) to predict post-surgical ISUP grade group. Of note, the best results in terms of prediction accuracy were reached with the combination of PET + ADC radiomic analysis (accuracy = 82 ± 5%). This combination of multi-modal radiomic analysis outperformed the accuracy of prostate biopsy for predicting the definitive post-surgical ISUP grade group. Analogous results were reported also by Ghezzo et al. [31], Yang et al. [26] (although in this paper, the authors considered ISUP grade group obtained either from biopsy or surgery as gold standard for training machine learning [ML] models), and Khateri et al. [25], confirming the high potential of ML models trained with radiomic features of staging PSMA PET to predict post-surgical definitive histology. Of note, the ML models proposed in the paper by Khateri and colleagues [25] were built by collecting imaging from three different institutions, performing a successful imaging harmonization through the ComBat algorithm [36]. This harmonization process enhanced the ML models’ performances and enables generalizability of the results of radiomic analysis. Finally, Wang et al. [32] performed a radiomic analysis of staging [^18^F]F-PSMA-1007 PET/CT in 161 PCa patients. The authors identified a radiomic signature significantly correlated to both high PSA values and aggressive ISUP grade group at surgery. That radiomic feature was combined with PSA values and evidence of metastatic disease at [^18^F]F-PSMA-1007 PET/CT to develop a nomogram for prediction of the ISUP grade group of PCa patients before radical prostatectomy. Of note, the radiomics nomogram demonstrated a high specificity (81.3%), slightly higher than that achieved by the radiomic analysis alone (78.1%).

In the work by Aksu et al. [33], the authors investigated the radiomic analysis on both early (immediately after radiotracer injection) and late (45 min after radiotracer injection) PSMA PET sets of images to predict the ISUP grade group. Considering early PSMA PET, Gray Level Run Length Matrix (GLRLM) was the best predictor at multivariate analysis, along with PSA. Conversely, considering the late PSMA PET dataset, SHAPE was the most relevant radiomic feature, again together with PSA values. The AUC, sensitivity, specificity, and accuracy were, respectively, 0.902, 76.2%, 84%, and 78.1% for the early images model and 0.924, 85.7%, 85%, and 85.4% for the late images model. Moreover, the same authors investigated Dmax, a novel parameter defined as the highest distance between any pathological findings. Dmax was strongly correlated with higher values of PSA and PSMA PET volumetric parameters (*p* < 0.001 each) and was higher in patients with ISUP grade group ≥ 3 (*p* = 0.023).

Finally, Ning et al. [23] elaborated five multiomics ML models in order to assess total-mount of Gleason Score (GS) grading and to compare them with the clinical standard of biopsy-derived GS in 65 PCa patients. They included in their models clinical, PET radiomics from [^68^Ga]Ga-PSMA-11 PET/MRI, genomics from exome sequencing, and pathomics features from immunohistochemical staining. Compared to the needle biopsy, among the five models, random forest showed higher AUC (87% vs. 75%), specificity (72% vs. 61%), positive predictive value (79% vs. 75%), and accuracy (78% vs. 77%), whereas sensitivity and negative predictive values were slightly lower (83% vs. 89% and 80% vs. 81%, respectively). In addition, among the 73 selected features, needle biopsy was that with higher mean permutation importance. The higher specificity of the model proposed could reflect in a better identification of low ISUP patients, while the high PPV may allow the selection of high-risk patients to provide timely treatments.

Other groups of authors tried to predict even more adverse pathologic features in PCa patients through radiomic analysis of staging PSMA-ligands PET imaging. Papp and colleagues [15] extracted radiomic features from [^68^Ga]Ga-PSMA-11 PET/MRI and combined them with clinical features (PSA and clinical tumor stage) to build ML models able to predict several adverse disease characteristics. The M_LH_ model—which was trained to discriminate high-risk vs. low-risk primary PCa—showed a AUC of 0.86, slightly higher than that obtained by standard [^68^Ga]Ga-PSMA-11 PET metrics, in analogy with the aforementioned studies. Similar results were published by Cysouw et al. [18] in a prospective cohort of 76 PCa patients, imaged before radical prostatectomy and pelvic lymph node dissection (PLND). ML models built with radiomic features extracted by [^18^F]F-DCFPyL PET/CT outperformed standard PET parameters to predict ISUP grade group ≥ 3, extra-capsular extension (ECE), lymph node involvement, and distant metastases (*p* < 0.01 each). Of note, the authors report that pre-processing methods did not significantly impact the performance of ML models. The same group of authors [24], insisting on the relevance of reproducibility and standardization of radiomics analysis, performed an internal and external validation of the previously published results in a second article. The models also maintained a high prediction accuracy at external validation, paving the way for their possible future application in daily clinical practice. ISUP grade group and ECE were among the disease features that Yao et al. [19] tried to predict with ML models built on radiomic features extracted from [^18^F]F-PSMA-1007, as well as vascular invasion. Interestingly, the authors performed various models, trained with radiomic features extracted by the same volume of interest (VOI) of the primary PCa, but using different thresholds of SUVmax (30%, 40%, 50%, and 60%). As a result, the 50% SUVmax ML model resulted the most accurate for prediction of both ISUP grade group (AUC = 0.82 and 0.80 in training and testing sets, respectively) and of vascular invasion (AUC = 0.74 both in training and testing sets). Conversely, ECE was better predicted by the ML model built using the 40% SUVmax (AUC = 0.74 both in training and testing sets). The prediction of ECE was also the aim of the study by Pan et al. [34]. The authors compared the radiomic analysis performed on multiparametric (mp) MRI with that of [^18^F]F-PSMA-1007 PET/CT images. Moreover, they used a third model built according to multimodal radiomics. The model with the best accuracy was that trained with mpMRI radiomics (AUC = 0.85 (0.78–0.90) and 0.74 (0.61–0.85) for training and test sets). The multimodal radiomic model outperformed the PET/CT model (0.73 (0.64–0.80) vs. 0.83 (0.75–0.89) at the training test and 0.62 (0.48–0.74) vs. 0.77 (0.64–0.87) at the test set; *p* < 0.01) but could not improve the accuracy of the mpMRI model. Similarly, Chan et al. [20] analyzed the voxel-wise relationship between [^68^Ga]Ga-PSMA-11 PET and mpMRI parameters and developed radiomics-based ML models for predicting both tumor location and local extension and tumor grade (high vs. low) in order to improve biologically targeted radiation therapy treatment planning. Overall, random forest classifier combining radiomics features from both PET and mpMRI detected intra-prostatic lesions better than single modalities, with a sensitivity, specificity, and AUC of 84%, 80%, and 89%, respectively. In addition, the tumor grading model showed promising results, with an AUC ranging between 67% and 99%.

Furthermore, Luo and colleagues [17] proposed ML models based on the radiomic analysis of [^18^F]F-PSMA-1007 PET/CT to predict seminal vesicle involvement. The authors performed a radiomic analysis according to two different segmentations: the first considering only the primary tumor with increased PSMA uptake within the prostate gland, performed semi-automatically using a 40% threshold of SUVmax; the second manually drawing a volume of interest (VOI) comprehending seminal vesicles (SV-VOI models). The SV-VOI random forest (RF) was the most accurate ML model for predicting seminal vesicle involvement, with an AUC = 0.96, significantly higher than that obtained by the reporting physician (AUC = 0.70).

Finally, the involvement of lymph nodes is an important variable affecting both clinical outcomes and patients’ management in different malignancies, including PCa [37]. Despite several studies having been published on the role of AI for detecting pathological lymph nodes using annotated datasets, Öğülmüş et al. [35] aimed to create a deep learning model to predict lymph nodes’ involvement in intermediate and high-risk PCa patients using the [^68^Ga]Ga-PSMA-11 PET/CT images, radiomics features and clinical variables. A model trained on 181 cases and tested on 48 cases was developed. Moreover, the model’s performance was compared with a reader study of the test data based on five radiation oncologists. Overall, the proposed AI model showed an efficacy with a mean accuracy of 85% associated with a low variability of only ±0.03%. In the reader study test set, accuracy was significantly higher for the AI model than physicians’ evaluations (accuracy 79% vs. 71%), opening the potential clinical application of AI model to more accurately differentiate between positive and negative lymph nodes.

### 3.4. Prediction of BCR and/or Prognosis

A few studies tried to predict the occurrence of BCR through the radiomic analysis of the primary PCa at staging PSMA PET. In the work by Papp et al. [15], ML_BCR_ and ML_OPR_ showed higher accuracy than conventional parameters to predict both biochemical recurrence (BCR) after radical prostatectomy (89% vs. 69%) and overall patient risk (OPR—91% vs. 70%). In the paper by Luo et al. [17], the authors investigated two main ML models built with radiomic analysis of pre-operative [^18^F]F-PSMA-1007 PET/CT—described in paragraph 3.3. to predict the risk of BCR. Each of the ML models proposed were able to stratify patients’ risk of BCR with high accuracy. In particular, the best model was the SV-VOI, which showed a hazard ratio (HR) of 2.36 (*p* = 0.022). Furthermore, the patients enrolled in the study were stratified in two groups—high-risk and low-risk of BCR—according to the two ML models. Of note, the progression free survival (PFS) rates were 63.4% and 14.9% for patients respectively defined as high-risk and low-risk according to the SV-VOI model (*p* < 0.001). Similarly, the PFS was significantly lower in patients defined as low-risk according to the 40%SUVmax-VOI model (58.5% vs. 23.9% vs. 58.5%; *p* < 0.001). However, it should be noted that only 47.1% of patients did not experience BCR shortly after radical prostatectomy (median follow-up: 4 months—range 1–33 months), limiting the generalizability of these results. Likewise, Li et al. [28] aimed to validate a clinical-radiomics model based on baseline [^18^F]F-PSMA-1007 PET/CT in order to predict the risk of BCR. The model was built on 236 patients and externally validated on 98 patients. Radiomics signature, based on three robust features after LASSO regression, showed a good performance with a C-index of 76% in the training cohort and 71% in the validation cohort, as well as remaining an independent predictor of BCR-free survival (HR: 2.48, *p* < 0.001). Moreover, the combination of clinical and radiomics signature improved the ability to predict BCR both in the training (C-index: 81%, *p* = 0.007) and in the validation cohort (C-index: 78%, *p* < 0.001).

On the other hand, Gülbahar Ates et al. [37] assessed the heterogeneity of the primary tumor to predict BCR in 51 patients who underwent [^68^Ga]Ga PSMA PET/CT before radiotherapy (n = 29) or surgery (n = 22). According to texture analysis, they identified INTENSITY-BASED-minimum grey level (*p* = 0.05), GLCM-sum variance (*p* = 0.019), and GLCM-cluster prominence (*p* = 0.05) as independent predictors of BCR at multivariate analysis.

Finally, a pivotal study by Bian et al. [27] aimed to predict the short term prognosis (i.e., PSA ≥ 0.1 ng/mL at 4–8 weeks after prostatectomy) according to the tumor microenvironment characterization, specifically investigating periprostatic adipose tissue, by pre-operative [^18^F]F-PSMA-1007 PET/CT. The radiomics score, consisting of 25 PET and CT features associated with clinical variables resulted in a model with optimal performance in the training, internal, and external validation cohorts, expressed with AUC of 85%, 77%, and 84%, respectively. As early persistence of elevated PSA levels is strictly correlated with long term prognosis, this dynamic nomogram could help clinicians to identify patients who will benefit from intensive follow-up. 

Finally, Yang et al. [21], combining [^68^Ga]Ga-PSMA-11 PET/CT and clinical factors, developed a ML model able to predict the ISUP grade group after radical prostatectomy in PCa patients with GS 1–2 tumors at biopsy. Of note, the authors used histopathology after RP standard of reference. The combined model, based on radiomics score and free-PSA/total-PSA ratio, showed higher accuracy than a single model, with an AUC in the training set and in the test set of 87.5% and 87.2%, respectively. This finding may help clinicians in achieving better patient stratification, which could enable a more accurate selection of patients as candidate for active surveillance, thus potentially reducing over- or under-treatment.

## 4. Discussion

PSMA PET has revolutionized the management of PCa, impacting on early definition of disease extension at staging [38]. Moreover, it has been shown to be the most accurate modality to identify the sites of relapse in BCR and to guide metastasis-directed therapy [8,39]. However, visual analysis and classic semiquantitative parameters (i.e., standardized uptake value—SUVmax—and PSMA tumor volume) have limitations and are not currently validated to reliably predict BCR [40]. Radiomic analysis of PSMA PET is being investigated for this purpose, similarly to what is being investigated in other solid tumors and with other radiotracers [41,42,43]. From this literature review, it emerged that the most promising and widely adopted data mining strategies are currently based on machine learning. In particular, algorithms such as Random Forest, Support Vector Machines, and Neural Networks are among the most commonly used classifiers. Furthermore, as shown in Table 3, there appears to be a certain level of standardization in the evaluation metrics used to assess model performance, with frequent reporting of indicators such as AUC, accuracy, sensitivity, and specificity. This trend is encouraging, as it facilitates greater comparability of radiomics and AI models across different studies. Recent evaluations of RQS indicate that a substantial proportion of radiomics studies report scores below 40% [44], suggesting limited adherence to the best practices and quality standards established by RQS. However, in this review, more than 57% of the studies employed a radiomics methodology that exceeded the 40% score threshold. Radiomic methodology still presents challenges related to reproducibility, generalizability, and clinical translation, primarily due to variability in imaging protocols, scanner parameters, and acquisition settings, which may introduce systematic biases. The extraction of radiomic features is influenced by image preprocessing, segmentation strategies, and feature computation techniques, potentially leading to variations across studies. As shown in Table 2, the most commonly used radiomic features in the analysis were shape features and second- and higher-order texture features. Shape features, which rely entirely on the accuracy of lesion segmentation, describe the anatomical extent and geometric properties of the tumor. Second-order features, such as those derived from the Gray-Level Co-occurrence Matrix (GLCM), characterize the spatial relationships between voxel intensities, capturing texture patterns related to homogeneity, contrast, and entropy. Higher-order features further extend this analysis by capturing more complex spatial arrangements and dependencies within the image. Although the biological significance of these more complex radiomic features is still not fully understood, the correlation of shape and first-order features with tumor heterogeneity, local invasion, and their potential association with disease stage and aggressiveness is well established in the literature.

Additionally, the high dimensionality of radiomic data, combined with limited sample sizes, poses a risk of overfitting, and may affect the statistical robustness of the predictive models. However, ongoing efforts to standardize imaging protocols will improve feature extraction techniques and model validation, progressively enhancing the reliability of the radiomic analyses. As shown in Table 3, only five studies performed external validation, which represents a significant limitation for the generalizability of the proposed models. Variability in scanners, acquisition protocols, and patient populations can substantially affect the robustness and reproducibility of radiomic features. These challenges highlight the necessity of prospective, multicenter study designs, as already emphasized. Fine-tuning models on small, non-representative datasets increases the risk of overfitting, potentially leading to models that demonstrate excellent performance metrics in development but lack clinical utility and fail to generalize to broader patient populations. The use of independent, multi-institutional datasets for external validation is increasingly recognized as a crucial step toward improving generalizability. In this perspective, some of the studies included in this review presented data harmonization strategies through multicenter datasets, paving the way for a solid and generalizable radiomic signatures. Advancements in methodological rigor, interpretability, and adherence to regulatory standards are expected to facilitate the successful clinical translation of radiomics, reinforcing its potential as a valuable tool in precision medicine.

In this literature review, we found high heterogeneity of the topics investigated with radiomic analysis of staging PSMA PET. Although we clustered the selected papers into three groups according to their objective of investigation, the outcomes measured were very heterogeneous and prevented us from conducting a meta-analysis. We identified that most of the papers currently published on this topic aimed to predict the definitive ISUP grade group through the radiomic analysis of baseline PSMA PET. mpMRI is currently considered the standard of care imaging modality for this purpose. However, mpMRI may yield false-positive and false-negative results in some circumstances, and high Prostate Imaging–Reporting and Data System (PIRADS) scores are not always correlated with high grade PCa [3]. Semiquantitative parameters at PSMA PET—in particular SUVmax—have been described as correlated to high ISUP grade, in analogy to other kinds of tumors [45,46,47]. However, these parameters are highly tomograph-dependent, limiting the possibility of defining universally validated cut-off values. Moreover, they represent only a very limited portion of the data that can be retrieved from PET imaging. Overall, the results published in the literature suggest that radiomic analysis could improve the pre-surgical characterization of the primary PCa contributing to the definition of the real ISUP grade group, which is different from that identified through prostate biopsy in a non-negligible percentage of cases [48]. This kind of use of radiomic analysis could be considered a modern “digital biopsy” to associate to the standard prostate biopsy for a deeper knowledge of each patient’s disease at diagnosis. Indeed, this additional information would improve the pre-therapeutic characterization of tumor aggressiveness. Multicenter trials are needed to validate and standardize the preliminary experiences currently published in the literature. In particular, the definition of a shared object of investigation would be of great relevance, since the articles identified in our review tried to predict different ISUP grade groups (i.e., ISUP grade group <3/>3 or <4/>4). Moreover, some studies investigated the potentialities of radiomic analysis applied to both PSMA PET and MRI [15,22,23,30,35]. Although it is too premature to perform a direct comparison of the results provided by radiomic analysis performed on these two imaging modalities, we can speculate that their combination may be synergistic. Future studies should also explore this perspective.

According to the results by Cysouw et al. [18], which were further validated in a second experience [24], ML models built on PSMA PET radiomic features could contribute to stratifying the risk of PCa patients before surgery. Several articles built artificial intelligence models that successfully predicted ECE [18,19,35] and seminal vesicles involvement [17], with good accuracy. The accurate definition of the local extension of the primary tumor is key for defining the best strategy when planning radical treatment. The use of these AI models in daily clinical practice, once validated, could contribute to a modern concept of personalized medicine, guiding treatment selection, intensification, or de-escalation.

Finally, despite improvements in imaging accuracy and in treatment selection and efficacy, some patients will inevitably experience BCR. The cumulative incidence of BCR is still ranging between 20–50% in patients undergoing radical treatment, with increasing risks in patients with higher D’Amico risk group [49,50,51]. Indeed, BCR is not always correlated to an increased risk of mortality; however, the evidence of increasing PSA values often leads to the prescription of imaging exams and of new treatments, in particular androgen deprivation therapy (ADT) [52]. Indeed, ADT impacts patients’ quality of life and increases the risk of major cardiovascular events [53]. Moreover, the evidence of rising PSA events impacts PCa patients’ emotional status, causing anxiety and leading to earlier and sometimes premature initiation of ADT [54]. Considering all these potential impacts of BCR on the management of PCa patients, the relevance of accurate imaging evaluation before radical treatment is clear. Some authors focused on the training of ML models able to predict the risk of BCR [17,27,28,37]. Bian et al. even proposed a new nomogram including pre-surgical [^18^F]F-PSMA-1007 radiomic features and clinical parameters to predict patients’ short term prognosis. Again, these results are still preliminary, although obtained in a cohort of 268 patients with a multicenter study design. However, they represent a relevant starting point for future experiences. In the last few years, a PSMA-ligands PET registry has been proposed to stratify PCa patients according to their survival outcomes [55]. We strongly believe that the incorporation of radiomic analysis in similar nomograms would further improve their prediction accuracy.

Lastly, some limitations of our systematic review should be underlined: first, most of the studies are retrospective and often derived from a single center; second, the wide heterogeneity among articles in terms of study design, textural parameters, and therapy schemes precludes our ability to perform any significant statistical analysis; third, many studies included in our review lacked external validation, which is fundamental to enhancing the clinical significance of radiomics and ML.

## 5. Conclusions

Our systematic review of the literature suggests that radiomic analysis of PSMA PET performed in the initial staging of patients with PCa could improve the characterization of tumor aggressiveness and the prediction of extra-capsular extension and seminal vesicles involvement. Moreover, AI models could contribute to predicting the risk of BCR after radical treatment. Indeed, characterization of tumor biology and early identification of risk recurrence are essential questions facing clinicians in planning therapeutic strategies. Therefore, the development of a radiomics nomogram model, incorporating both clinical and radiomics signatures, could potentially be useful in clinical practice for proving personalized therapeutic schemes.

## Figures and Tables

**Table 1:** Summary of general characteristics of studies published in the literature.

Author	Year	Study Design	N. pts	Radiotracer Imaging Modality of Radiomic Analysis	Aim	Standard of Reference	Main Results
Papp et al. [15]	2020	P	52	[^18^F]F-choline and [^68^Ga]Ga-PSMA-11PET/MRI	Predict GS (≥4 vs. <4), BCR after RP, and OPR	Histopathology after RP	ML models showed better accuracy than conventional PET parameters used in daily clinical practice to predict ISUP grade group, BCR, and OPR
Zamboglou et al. [16]	2019	P	20	[^68^Ga]Ga-PSMA-11PET	Predict ISUP grade group (≥ 3 vs. <3) and lymph node involvement	Histopathology after RP	GLSZM was able to accurately predict ISUP grade group and pN0 vs. pN1.
Luo et al. [17]	2024	R	140	[^18^F]F-PSMA-1007PET/CT	Predict seminal vesicle invasion and BCR after RP	Histopathology after RP	The ML models accurately predicted seminal vesicle involvement and stratified PCa patients according to their risk of BCR after RP.
Cysouw et al. [18]	2021	P	76	[^18^F]DCFPyLPET/CT	Predict ISUP grade group (≥3 vs. <3), ECE, lymph node involvement, and metastatic disease	Histopathology after RP	ML models outperformed conventional PET parameters for risk stratification of PCa patients
Yao et al. [19]	2022	R	173	[^18^F]F-PSMA-1007PET	Predict ISUP grade group, ECE, and VI	Histopathology after RP	[^18^F]F-PSMA-1007 PET-based radiomics features at 40–50% SUVmax were able to predict multiple PCa biological features
Chan et al. [20]	2023	P	19	[^68^Ga]Ga-PSMA-11PET and mpMRI	Predict tumor location and grade	Histopathology after RP	ML models, based on PET and mpMRI, differentiated well between low- and high-risk PCa
Yang et al. [21]	2024	P	75	[^68^Ga]Ga-PSMA-117PET/CT	Predict adverse pathology in patients with biopsy GS 1–2	Histopathology after RP	The combined model, radiomics + PSA ratio, was superior to the single model for stratifying GS 1–2 patients.
Feliciani et al. [22]	2022	R	28	[^68^Ga]Ga-PSMA-11PET and mpMRI	Predict ISUP grade group (1 vs. ≥2)	Histopathology after RP	Radiomics extracted from both MRI-ADC and [^68^Ga]Ga-PSMA-11 PET could predict ISUP grade group.
Ning et al. [23]	2024	P	65	[^68^Ga]Ga-PSMA-11PET/MRI	Assess total GS grading compared to biopsy-derived GS	Histopathology after RP	Random forest was superior to biopsy alone GS in terms of AUC, accuracy, specificity, and NPV.
Luining et al. [24]	2023	R	72	[^18^F]DCFPyLPET	Validate the results obtained in the previous original article [Cysouw et al.] [18] through a multicenter experience	Histopathology after RP	The models maintained a high prediction accuracy at external validation to discriminate high-risk vs. low-risk PCa
Khateri et al. [25]	2024	R	90	[^68^Ga]Ga-PSMA-11PET	Build ML models to predict GS (≤7 vs. >7) using imaging acquired in three different institutions	Histopathology after RP	ML models could accurately predict post-surgical ISUP grade group. ComBat harmonization algorithm enhanced the models’ performances and enables inter-center generalizability.
Yang et al. [26]	2024	R	356	[^18^F]F-PSMA-1007PET/CT	Build ML models to predict ISUP grade group (<4 vs. ≥4)	Histopathology at prostate biopsy or after RP	ML models built on radiomic analysis outperformed the clinical model to predict ISUP grade group.
Bian et al. [27]	2024	R	268	[^18^F]F-PSMA-1007PET/CT	Predict short term prognosis, based on periprostatic adipose tissue assessment	Histopathology at prostate biopsy or after RP	The radiomics-clinical combined model demonstrated an optimal performance in terms of AUC.
Li et al. [28]	2024	R	236	[^18^F]F-PSMA-1007PET	Predict BCR after RP	BCR after RP	PET-based clinical-radiomics model showed high predictive performance.
Öğülmüş et al. [29]	2025	R	229	[^68^Ga]Ga-PSMA-11PET/CT	Predict lymph nodes metastases	PET visual analysis	The AI model outperformed the reader’s analysis.
Solari et al. [30]	2021	R	101	[^68^Ga]Ga-PSMA-11PET/MRI	Predict ISUP grade group (≥3 vs. <3)	Histopathology after RP	The combination of PET + ADC radiomic analysis outperformed the prostate biopsy in terms of prediction accuracy of post-surgical ISUP grade group.
Ghezzo et al. [31]	2023	R	47	[^68^Ga]Ga-PSMA-11PET	Build ML models to predict ISUP grade group (<4 vs. ≥4)	Histopathology after RP	All radiomics-based ML models trained with at least two RFs outperformed the control models.
Wang et al. [32]	2022	R	161	[^18^F]F-PSMA-1007PET/CT	Combine clinical and PSMA PET/CT radiomic features to build a nomogram for prognostic stratification of PCa patients	Histopathology at prostate biopsy or after RP	A radiomic signature identified was significantly correlated to both PSA values and ISUP grade group. The radiomics nomogram demonstrated a higher specificity (81.3%) than the radiomics features alone (78.1%).
Aksu et al. [33]	2022	R	41	[^68^Ga]Ga-PSMA-11PET	Predict ISUP grade group (≥3 vs. <3) according volumetric and radiomic analysis of early and late PSMA PET; investigate the relationship between Dmax obtained in early PET images and histopathology and PSA.	Grading at prostate biopsy	Some radiomic features extracted from both early and late PSMA PET images accurately predicted the ISUP grade group. Dmax was strongly correlated with higher values of PSA and PSMA PET volumetric parameters and was higher in patients with ISUP grade group ≥ 3
Pan et al. [34]	2024	R	197	[^18^F]F-PSMA-1007PET/CT and mpMRI	Predict ECE with multimodal radiomic analysis	Histopathology after RP	The mpMRI radiomic model was the most accurate for predicting ECE. The multimodal radiomic model outperformed the PET/CT model but did not improve the accuracy of the mpMRI model.
Gülbahar Ates et al. [35]	2024	R	51	[^68^Ga]Ga-PSMA-11PET	Predict BCR in patients who underwent RT or RP	BCR after RP or RT	INTENSITY-BASED-minimum grey level and GLCM-sum variance were independent predictors of BCR.

Abbreviations: AI = artificial intelligence; AUC = area under the curve; BCR = biochemical recurrence; ECE = extracapsular extension; GLSZM = gray level size zone matrix; GS = Gleason Score; ISUP = International Society of Urological Pathology; ML = machine learning; mpMRI = multiparametric magnetic resonance imaging; MRI = magnetic resonance imaging; NPV = negative predictive value; NR = not reported; OPR = overall patient risk; P = prospective; PET/CT = positron emission tomography/computed tomography; PET/MRI = positron emission tomography/magnetic resonance imaging; PSA = prostate specific antigen; R = retrospective; RF = radiomic features; RP = radical prostatectomy; RT = radiotherapy; VI = vascular invasion.

**Table 2:** Summary of the studies’ radiomic features analyses.

Author	Segmentation Method (Algorithm)	Segmentation SW (Class)	Radiomics FTs Type (n)	Selected FTs	Radiomic SW (Class)	Statistical Analysis to Reduce Redundant Variables	RQS 2.0 (%)
Papp et al. [15]	Semi-automatic (standard three-dimensional iso-count VOIs)	Hybrid 3D V4.0.0 (C)	shaped-based first-order second or higher order (GLCM, GLSZM, GLRLM, NGLDM, GLDZM) (FTs n = 446)	80	MUW Radiomics Engine V2.0 (IH)	Covariance matrix analysis, Pearson correlation coefficient	27 (40.91%)
Zamboglou et al. [16]	Semi-automatic (WL 0–5 SUV, threshold of 40% of SUVmax, coregistration of the histopathology with PET image)	MITK V2016.11 (OS) 3D-Slicer V4 (OS)	first order second or higher order (GLCM, GLRLM, GLSZM, NGTDM, WBFP) (FTs n = 133)	131	MATLAB (C)	Wilcoxon Rank test, Spearman correlation coefficient	30 (45.45%)
Luo et al. [17]	Manual and semi-automatic (threshold 40% SUVmax)	uAI Research Portal (C)	shaped-based first-order second or higher order (GLCM, GLSZM, GLRLM, GLDM, NGTDM) (FTs n = 2264)	PET:20 CT:11	uAI Research Portal (C)	Relief, SelectKBest and LASSO	21 (31.82%)
Cysouw et al. [18]	Semi-automatic (region-growing algorithm with a background adapted peak threshold)	N.A.	shaped-based first-order second or higher order (GLCM, GLRLM, GLSZM, GLDZM, NGTDM, NGLDM) (FTs n = 480)	48	RaCaT (OS)	PCA, RF, ANOVA	23 (34.85%)
Yao et al. [19]	Semi-automatic (thresholds 30%, 40%, 50%, and 60% SUVmax)	LIFEx V6.30 (OS)	shaped-based first-order second or higher order (GLCM, GLRM, NGLDM, GLZLM) (FTs n = 70)	10	LIFEx V6.30 (OS)	ICC, mRMR	23 (34.85%)
Chan et al. [20]	Semi-automatic (guided by histology)	3D-Slicer	Shape features first-order second or higher order (GLCM, GLRLM, GLSZM, NGTDM, GLDM) Wavelet, GM, LoG, LBP (FTs n = n.d.)	30	Python (OS)	-reject all highly correlated features -retain the top 10% of the features based on the ANOVA test -retain the top 50 features based on the mean decrease in random forest Gini impurity.	26 (39.39%)
Yang et al. [21]	Manual	3D-Slicer V5.3.0	shaped-based first-order second or higher order (GLCM, GLDM, GLRLM, GLSZM, NGTDM) (FTs n = 107)	6	Python V3.7.4 (OS)	mRMR, LASSO	30 (45.45%)
Feliciani et al. [22]	Manual for MR imaging; semi-automatic for PET imaging (threshold SUV(bw)max of 3 g/mL)	Watson Elementary for MR imaging (C) MIM Maestro for PET imaging (C)	first order, second or higher order (GLCM, GLRM) (FTs n = 218)	PET: 29 MRI-ADC:87	SOPHiA DDM™ (C)	ICC, LASSO	29 (43.94%)
Ning et al. [23]	Manual	N.A.	Shape-based first-order second or higher order (GLCM, GLRLM, GLSZM, GLDM, NGTDM) (FTs n = 203)	57	Python (OS)	mRMR	29 (43.94)
Luining et al. [24]	Semi-automatic (region growing with threshold 50%, 55%, 60%, 65%, and 70% SUVpeak)	ACCURATE tool (OS)	shaped-based first-order second-order or higher order (FTs n = 480)	184	RaCaT (OS)	PCA, RFE, univariate feature selection, LASSO	33 (50.00%)
Khateri et al. [25]	Manual	LIFEx V7.0.0	first order second or higher order (GLCM, GLRLM, NGLDM, GLZLM) (FTs n = 69)	30	Python (OS)	mRMR, ANOVA, KW, Relief	42 (63.64%)
Yang et al. [26]	Manual and automatic (DL tool Total- Segment)	LIFEx V7.3.0	shaped-based first-order second or higher order (GLCM, GLRLM, NGTDM, GLSZM) (FTs n = 215)	134	Python (OS)	LASSO, RFE, REIF, MUIF, mRMR, IFGN	41 (62.12%)
Bian et al. [27]	Semi-automatic	3D Slicer V4.11	shaped-based first-order (GLCM, NGLDM, GLZLM, GLRLM) FTs n = n.d.	25	LIFEx V6.30	mRMR, LASSO	33 (50.00%)
Li et al. [28]	Manual	LIFEx V7.3.0	shaped-based first-order second or higher order (GLCM, GLSZM, GLRLM, NGTDM) (FTs n = 124)	3	LIFEx V7.3.0	ICC, LASSO	33 (50.00%)
Öğülmüş et al. [29]	Automatic (DL)	Python	shape Features first-order second or higher order (GLCM, GLRLM, GLSZM, GLDM, NGTDM) (FTs n = 105)	n.d.	Python (OS)	DL model	* 41 (67.21%)
Solari et al. [30]	Automatic on PET images (FLAB); manual on MR images	FLAB segmentation tool (IH)	shaped-based first-order second or higher order (GLCM, GLSZM, LRLM, NGTDM, and GLDM) (FTs n = 107)	T1w: 9 T2w: 7 ADC: 7 PET: 9 PET + T1w: 10 PET + T2w: 7 PET + ADC: 9	N.A.	RFE	22 (33.33%)
Ghezzo et al. [31]	Manual	3D-Slicer V29	shaped-based first-order second or higher order (GLCM, GLSZM, GLRLM, NGTDM, GLDM) (FTs n = 103)	4	ComBat SW	mRMR	25 (37.88%)
Wang et al. [32]	Semi-automatic (threshold 40% of SUVmax)	ITKSNAP V3.8 (OS)	shaped-based first-order second or higher order (GLCM, GLSZM, GLRLM, NGTDM, GLDM) Wavelet, LoG, GFF (FTs n = 944)	30	Philips Radiomics Tool (C)	LASSO	29 (43.94%)
Aksu et al. [33]	Semi-automatic (PSMA uptake above 2.5 SUV)	LIFEx (OS)	shaped-based second order or higher order (GLCM, NGLDM, GLRLM, GLZLM) (FTs n = 41)	36	LIFEx (OS)	Spearman correlation, Mann-Whitney U test	20 (30.30%)
Pan et al. [34]	Manual and semi-automatic (threshold 40% SUVmax)	ITKSNAP V3.6 (OS)	shape features first-Order second or higher order (GLCM, GLDM, GLRLM, GLSZM, NGTDM) (FTs n = 1316) ** shape features first-Order second or higher order (GLCM, GLRLM, NGLDM, GLZLM) (FTs n = 140) ***	20	LIFEx V6.3	mRMR, LASSO	24 (36.36%)
Gülbahar Ates et al. [35]	Semi-automatic (threshold 40% SUVmax)	LIFEx	shaped-based first-order second or higher order (GLCM, GLRLM, NGTDM, GLSZM) (FTs n = n.d.)	3	LIFEx V7.3	univariate and multivariate analysis	24 (36.36%)

* evaluated in DL type. ** from mpMRI. *** from PET/CT. DL: Deep Learning; FLAB: Fuzzy-Logically Adaptive Bayesian; FTs: features; GFF: gradient filtered features; GLCM: gray-level co-occurrence matrix; GLDM: gray-level difference matrix; GLRLM: gray-level run-length matrix; GLSZM: gray-level size zone matrix; GLZLM: gray-level zone-length matrix; GM: gradient magnitude; HGZE: high gray-level zone emphasis; ICC: intra-class correlation; IFGN: Information Gain; KW: Kruskal-Wallis; LASSO: least absolute shrinkage and selection operator; LBP: local binary pattern; LoG: Laplacian of Gaussian; N.A.: not available; n.d.: not defined; MUIF: Mutual Information Feature; MUW: Medical University Wien; NGLDM: neighborhood gray-level different matrix; NGTDM: neighborhood gray-tone difference matrix; OS: open source; PCA: principal component analysis; REIF: Relevance and Information Filtering; RFE = Recursive Feature Elimination; RQS: radiomics quality score; SW: software.

**Table 3:** Summary of machine learning studies’ data mining.

Authors	AI SW (Class)	Data-Mining Methods	Training/Test Set (n. of Patients)	Validation (n. of Patients)	Performance Score	Clinical Application
Papp et al. [15]	N.A.	RF	52	Internal (52)	AUC	N.A.
Zamboglou et al. [16]	R V.3.4.4 (OS) SPSS V24 (C)	LR	20	Internal (40)	AUC, ROC	N.A.
Luo et al. [17]	uAI Research Portal IBM SPSS V25.0 (C)	LR, RF, SVM	112/28	N.A.	AUC, SEN, SPEC, ACC	N.A.
Cysouw et al. [18]	Python V3.6 (OS)	RF	61	Internal (15)	AUC	N.A.
Yao et al. [19]	IBM SPSS V25.0 (C) R V4.0.2 (OS)	SVM with RBF kernel	122/51	N.A.	AUC, ACC, SEN, SPE, F1Score, NRI	N.A.
Chan et al. [20]	Python (OS)	RFC, SVC	19	N.A.	AUC, ROC, SEN, SPE	N.A.
Yang et al. [21]	IBM SPSS V26.0 (C) R (OS)	LR	52	Internal (23)	AUC, SEN, SPE, PPV, NPV, Radscore	N.A.
Feliciani et al. [22]	R,RStudio (OS)	LR	19/9	N.A.	AUC, ROC	N.A.
Ning et al. [23]	Python (OS)	kNN, RF, XGBoost, SVM, LR	45	Internal (20)	AUC, SPE, ACC, PPV, NPV, SEN	N.A.
Luining et al. [24]	Python V3.7 (OS)	RF, LR	72/24	External (27)	AUC, ROC, SEN, SPE	nomogram
Khateri et al. [25]	Python (OS)	LR, KNN, ET, LDA, RF	62/16	External (12)	AUC, ROC, PREC, ACC, REC, F1Score	N.A.
Yang et al. [26]	Python (OS)	LR, RF, SVM, GBDT, and XGBoost	241	External (115)	AUC, ROC, bACC	DC
Bian et al. [27]	IBM SPSS V25.0 (C) R V4.0.2 (OS)	LR	156/65	External (47)	AUC, ROC, Radscore, NRI	Nomogram, DC
Li et al. [28]	R V4.1.1 (OS)	univariate and multivariate Cox regression analysis	236	External (98)	AUC, C-index	Nomogram, DC
Öğülmüş et al. [29]	Python V3.9 (OS)	DL	181/48	N.A.	AUC, ROC, ACC, PREC, REC, F1 Score, MCC	N.A.
Solari et al. [30]	Python (OS)	SVM with RBF and a “one-vs-rest” multi-class approach	67 + 53 *	Internal (34)	bACC, SEN, SPE	N.A.
Ghezzo et al. [31]	Python V3.7 (OS)	LR, SVM, KNN	50	Internal (10)	bACC, SEN, SPE, PPV, NPV	N.A.
Wang et al. [32]	R V4.0.2 (OS) IBM SPSS V13.0 (C)	LR	112	Internal (49)	AUC, ROC, NPV, PPV	nomogram
Aksu et al. [33]	IBM SPSS V28.0 (C)	LR	N.A.	N.A.	AUC, ROC	N.A.
Pan et al. [34]	IBM SPSS V25.0 © R V4.0.2 (OS)	LR	139/58	N.A.	AUC, ACC, SEN, SPE, NPV, PPV, NRI	N.A.
Gülbahar Ates et al. [35]	IBM SPSS V25.0 (C)	Cox regression analysis	51	N.A.	Youden’s index, ROC	Kaplan-Meier

* SMOTE oversampling. ACC: accuracy; AUC: area under the curve; bACC: balanced accuracy; C-index: Harrel’s concordance index; DC: decision curves; DL: deep learning; ET: Extra Trees; IH: in-house; GBDT: Boosting Decision Tree; KNN: K-nearest neighbors; LDA: linear discriminant analysis; LLM: Logic Learning Machine; LR: linear regression; MCC: Matthews Correlation Coefficient; ML: machine learning; N.A.: Not Available; NPV: negative predictive value; NRI: Net reclassification improvement; PPV: positive predictive value; PCS: precision; NPV: negative predictive value; RADSCORE: radiomics score; RBF: Radial basis function; REC: recall; RF: random forest; RFC: random forest classifier; ROC: Receiver Operating Characteristic; SVC: Support Vector Classifier; SEN: sensitivity; SPE: specificity; SVC: Support Vector Classifier; SVM: support vector machines; XGBoost: eXtreme Gradient Boosting.

## Data Availability

No new data were created.

## Supplementary Materials

The following supporting information can be downloaded at: https://www.mdpi.com/article/10.3390/jcm14103318/s1, Table S1: PRISMA checklist.
